# Transcriptomic response of *Daphnia magna* to nitrogen‐ or phosphorus‐limited diet

**DOI:** 10.1002/ece3.7889

**Published:** 2021-07-16

**Authors:** Zhimeng Xu, Yingdong Li, Meng Li, Hongbin Liu

**Affiliations:** ^1^ SZU‐HKUST Joint PhD Program in Marine Environmental Science Shenzhen University Shenzhen China; ^2^ Department of Ocean Science The Hong Kong University of Science and Technology Kowloon China; ^3^ Shenzhen Key Laboratory of Marine Microbiome Engineering Institute for Advanced Study Shenzhen University Shenzhen China; ^4^ Southern Marine Science and Engineering Guangdong Laboratory (Guangzhou) Guangzhou China; ^5^ Hong Kong Branch of Southern Marine Science and Engineering Guangdong Laboratory The Hong Kong University of Science and Technology Hong Kong China

**Keywords:** *Daphnia magna*, homeostasis, nutrient limitation, phenotypic performance, transcriptome

## Abstract

1. Effects of nutrient‐imbalanced diet on the growth and fitness of zooplankton were widely reported as key issues to aquatic ecology. However, little is known about the molecular mechanisms driving the physiological changes of zooplankton under nutrient stress.
2. In this study, we investigated the physiological fitness and transcriptomic response of *Daphnia magna* when exposed to nitrogen (N)‐limited or phosphorus (P)‐limited algal diet (*Chlamydomonas reinhardtii*) compared to regular algae (N and P saturated).
3. *D. magna* showed higher ingestion rates and overexpression of genes encoding digestive enzymes when fed with either N‐limited or P‐limited algae, reflecting the compensatory feeding. Under P‐limitation, both growth rate and reproduction rate of *D. magna* were greatly reduced, which could be attributed to the downregulated genes within the pathways of cell cycle and DNA replication. Growth rate of *D. magna* under N‐limitation was similar to normal group, which could be explained by the high methylation level (by degradation of methionine) supporting the body development.
4. Phenotypic changes of *D. magna* under nutrient stress were explained by gene and pathway regulations from transcriptome data. Generally, *D. magna* invested more on growth under N‐limitation but kept maintenance (e.g., cell structure and defense to external stress) in priority under P‐limitation. Post‐translational modifications (e.g., methylation and protein folding) were important for *D. magna* to deal with nutrient constrains.
5. This study reveals the fundamental mechanisms of zooplankton in dealing with elemental imbalanced diet and sheds light on the transfer of energy and nutrient in aquatic ecosystems.

## Graphical abstract

In this study, we investigated the physiological fitness and transcriptomic response of *Daphnia magna* when exposed to nitrogen (N)‐limited or phosphorus (P)‐limited algal diet. We attributed the changes in ingestion, growth, and reproduction rates of *D. magna* to the different gene regulation mechanisms, explaining their trade‐off strategies to deal with nutrient constrains.

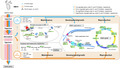

## 1 INTRODUCTION

Ecological stoichiometry, a branch of ecology that considers how the balance of energy and elements influences the living systems (Sterner & Elser, 2002), has been widely studied in both land and aquatic systems where stoichiometric imbalances between predator and prey can affect trophic interactions and ecosystem functions. Elemental compositions (e.g., C:N:P, short for carbon:nitrogen:phosphorus) of phytoplankton can vary easily and frequently following hydrographic conditions of ambient water. Nutrient‐limited algae usually are deficient in certain constituents (e.g., certain lipids and amino acids) and are considered as low‐quality prey for herbivorous zooplankton (lack of food preference; Weers & Gulati, 1997). The performances (e.g., ingestion, growth, and reproduction rates) of zooplankton in response to low‐quality diet, which have consequences for both phytoplankton and zooplankton population dynamics and energy flow, are important to the understanding of ecological stoichiometry in aquatic systems.

“All life is chemical,” and certain chemical elements are critical to organisms for building and maintaining basic biological structures and core functions (Jeyasingh et al., 2011; Sterner & Elser, 2002). Transcriptomics (expressed mRNA) can be used as a tool to understand the molecular mechanisms underlying many biological processes related closely to ecosystem functions. It can not only explain the physiological responses of organism under environmental stresses, but also provide a global expression pattern which may lead to a more detailed investigation of some “key genes” (Wang et al., 2009). However, transcriptomic studies are much less applied to the ecology of zooplankton (Lenz et al., 2021), compared with other organisms such as bacteria, phytoplankton, land plants, and vertebrates (Hua et al., 2004; Nguyen et al., 2015).

Daphnids, which filter particles in the size range of 0.5–50 μm including algae and bacteria, are key aquatic herbivores and model species to study aquatic ecology. Daphnids may often experience nutrient‐limited diet coming from (1) intracellular biochemical difference among ingested food, for example, green eukaryotic algae, cyanobacteria, and fungi, (2) movement of daphnids between heterogeneous food patches, and (3) seasonal succession in the taxonomic composition of phytoplankton (Koussoroplis et al., 2017). Much work has been done to study the phenotypic response of daphnids, especially on the growth and reproduction rates, to the nutritional constraints induced by diet, which showed that food elemental compositions (particularly N and P) have profound effects on daphnids’ population dynamics. For instance, P‐deficient food can significantly reduce the growth rate and egg production of daphnids, and the growth rate of daphnids correlates well with algal P content (Sterner et al., 1993; Weers & Gulati, 1997). N‐limited diet can also affect the performance of daphnids, but the negative effects were not as severe as P‐limited algae in some studies (Sterner et al., 1993; Weers & Gulati, 1997). However, except two studies reporting the transcriptomic response of daphnids to P‐limitation using gene microarrays (Jeyasingh et al., 2011; Roy Chowdhury et al., 2015), the metabolic mechanisms (in terms of gene regulation and involved pathways) of daphnids’ physiological changes in response to nutrient limitation, especially the different responses between N‐ and P‐limitation, remain unclear.

In this study, we sequenced the mRNA extracted from *Daphnia magna* (one kind of daphnids species widely distributed in fresh water) fed by N‐ or P‐limited green algae (*Chlamydomonas reinhardtii*) for 7 days and compared that to those on normal diet (N‐ and P‐sufficient *C*. *reinhardtii*). Through gene and pathway enrichment analysis, we found that *D. magna* upregulated the expression of genes encoding digestive enzymes under both N‐limited and P‐limited diets, which explains their higher ingestion rate and compensatory feeding behavior. Their growth and reproduction were severely impaired under P‐limitation, which can be attributed to the downregulation of genes controlling cell cycle and DNA replication. Under N‐limitation, gene regulation related to methionine metabolism maintained the body growth but reduced reproduction of *D. magna*. Our results suggest that post‐translational modifications (e.g., methylation and protein folding) were important for *D. magna* to deal with nutrient imbalance and stress.

## 2 METHODS

### 2.1 Culture of algae

Green algae *C*. *reinhardtii* (CC1690) were grown in 1‐L vessels with BG11 medium (Stanier et al., 1971). P‐limited algae were obtained by reducing K_2_HPO_4_ by 10‐fold from 40 to 4 mg/L, while N‐limited algae were obtained by reducing the concentration of NaNO_3_ by 30‐fold from 1.5 to 0.05 g/L. No modification of medium was made for the normal group. Algae were cultured in a chamber under constant temperature of 23.5℃ and a 14:10 light/dark cycle (20 μmol/m^2^ s^−1^). Concentration of algae was measured daily by a Beckman Coulter Z2 Cell and Particle Counter (Beckman Coulter Inc.). Cellular carbon (C) and nitrogen (N) of algae in three conditions (i.e., normal, N‐limited, and P‐limited) were quantified by a FlashSmart CHNS Elemental Analyzer (Thermo Fisher). Cellular P was analyzed as orthophosphate after acidic oxidative hydrolysis with 1% HCl. The concentration of PO_4_^3−^ was measured manually according to a previous report (Strickland, 1968).

### 2.2 Measurement of ingestion, growth, and reproduction rates of *D. magna*

Before this experiment, *D. magna* was cultured in the creek water and fed with sufficient *C. reinhardtii*. During the experiment, newborns of *D. magna* were collected within 12 hr after birth and transferred to 3 glass beakers (volume = 5 L) with 0.45 μm filtered fresh water. Three kinds of algae (i.e., N‐limited, P‐limited, and nutrient‐sufficient *C*. *reinhardtii* as described above) were added to each beaker, respectively, as prey for *D. magna*. Ingestion rate, growth rate, and reproduction rate (numbers of egg in the brood and daily newborns) of *D. magna* in each treatment were measured on the 1st, 3rd, 5th, and 7th days during the experiment.

For ingestion rate measurement, on each sampling day, 10 individuals of *D. magna* were taken from the beaker and put in a 600‐ml polycarbonate bottle (Nalgene), with three replicates. Algae concentration (as food) was kept constantly to 100,000 cells/ml (i.e., ~2.5 mg C L^−1^) which was above the incipient limiting level according to a pre‐experiment. Grazing experiment was conducted for 24 hr, and ingestion rate (*I*, cells daphnids^−1^ day^−1^) was calculated following the previous studies (Frost, 1972):

$$I=\ln\left(Ct^{\prime }/Ct\right)\times \left(V/nt\right)\times \left[C\right]$$

where $Ct^{\prime }$ and *Ct* (cells ml^−1^) are the prey (*C*. *reinhardtii*) concentrations at the end of incubation in control and experimental bottles, respectively. *V* is the volume of the culture (ml), *t* is the incubation time (day), and *n* is the number of *D. magna* used. *C* is the prey concentration in the experimental bottles averaged over the incubation period. Prey (fixed by acidic Lugol's solution) concentration was counted using an inverted microscope (Olympus CK30).

To measure growth rate, on each sampling day, 15 individuals of *D. magna* were randomly taken from the beaker for each treatment. Body length was measured from the anterior margin of the eye to the base of the tail spine of *D. magna* using a microscope (Olympus IX51). Daily increase in body length was used to represent growth rate.

To measure reproduction rate, 10 individuals of *D. magna* (with triplicates for each treatment) were taken from the beaker on the first day and cultured in a 600‐ml polycarbonate bottle (Nalgene) for 7 days with the same medium in the beaker. On each sampling day (i.e., 1st, 3rd, 5th, and 7th days), a number of eggs and newborns in the brood pouch of *D. magna* were calculated using an inverted microscope (Olympus CK30). Newborns were removed immediately after calculating. Daily increase in egg production and newborns was used as reproduction rate.

### 2.3 RNA extraction

On the 7th day, for each type of diet, 50 individuals (rinsed by Milli‐Q water for three times. No starvation step was conducted as it will cause extra transcriptomic response) were taken from the beaker and pooled as one sample. Triplicate samples were collected for each type of diet. Body tissues were grinded manually in a PCR tube and kept in RNAlater solution at −80℃. RNA was isolated using TRIzol reagent (Takara) and RNA Mini kit (Thermo Fisher) in accordance with the manufacturer's instructions. Genomic DNA was removed using the Turbo DNA‐free DNase kit (Ambion), and mRNA was isolated with the MicroPoly (A) Purist kit (Ambion). RNA integrity was evaluated using an Agilent 2100 bioanalyzer (Agilent Tech.).

### 2.4 Sequencing and transcript assembly

Each replicate mRNA was sequenced with Illumina HiSeq2500 (Novogene), generating 150‐bp paired‐end reads. Raw reads were processed sequentially by quality control, reads filtration, assembly, gene prediction, quantification, and sorting. FastQC (Babraham bioinformatics) was used to assess the quality of raw reads and quality control (length >140 bp, without ambiguous “N,” and average base quality >20; Andrews, 2010). SRC_c method (Meng et al., 2018) was employed to sort reads affiliated to *D. magna* from the whole metatranscriptome (mainly include reads from *D. magna*, bacteria, fungi, and *C. reinhardtii*) with indexed *k*‐mers set to 32 and a suggested default similarity value (50%). For this step, library of *D. magna* was built using RNA‐seq datasets from a previous study (Orsini et al., 2016). Then, *D. magna* reads were assembled to contigs using Trans‐ABySS v2.0.1 with k‐mer set from 32 to 92, with a step of five (Robertson et al., 2010). Open reading frames (ORFs) were predicted from the assembled contigs by TransDecoder v5.3.0 with minimum length set to 100 amino acid (Haas et al., 2013). The coverage information of predicted genes was revealed by mapping short reads to ORFs using Bowtie v2.2.9 (Langmead & Salzberg, 2012) and SAMtools v1.9 with default similarity setting (Li et al., 2009). Lastly, the MEGAN software (Community Edition, v. 6.15.1; Huson et al., 2016) was used as a second step to sort the genes affiliated to *D. magna* (database file: “prot‐acc2tax‐Jul2019X1.abin”).

### 2.5 Differential expression gene analysis, GO, and KEGG enrichment

Differentially expressed genes (DEGs) were estimated using “edgeR” in the “Bioconductor” package in R (v 3.4.1; Robinson et al., 2010). Gene showing |log_2_(fold change)| > 1 and *p*‐value < .05 (quasilikelihood *F* test) at any of the two comparison groups with triplicated gene expression data (N‐limitation vs. normal and P‐limitation vs. normal) was defined as DEG (Lyu et al., 2019). DEGs were further annotated by Gene Ontology (GO) and Kyoto Encyclopedia of Genes and Genomes (KEGG) using DIAMOND (v0.9.21.122) with the following parameters: blastp; *k* parameter = 1; *e*‐value = 10^–7^ (Buchfink et al., 2015). Higher level of GO terms and KEGG pathways were enriched by DEGs using TBtools (Chen et al., 2020), and corrected Benjamini *p*‐value (i.e., *q*‐value) < .05 as cutoff was used to define significantly enriched KEGG pathways.

### 2.6 Validation of RNA‐seq by qPCR

To validate the RNA‐seq results, 7 DEGs, mainly within the enriched KEGG pathways, were randomly selected to confirm the expression profiles in our study. Primers (final concentration = 10 mM) were designed by Primer Premier v6.00. For each sample, 500 ng of extracted RNA was used for reverse translation by HiScript III RT SuperMix for qPCR kit (Vazyme Biotech). After the synthesis of cDNA, qPCR was performed with FastStart Universal SYBR Green Master (Roche) on LightCycler 384 (Roche). The qPCR followed an initial hold at 50℃ for 2 min and 95℃ for 10 min followed by 45 cycles of 95℃ for 15 s and 60℃ for 1 min. The relative abundances of genes were calculated by a 2^−ΔΔCt^ method, with β‐actin as an internal control (Lyu et al., 2019).

### 2.7 Statistical analysis

Statistical analysis was conducted in R software (v 4.0.0). Welch two sample *t* test was used to test the significant differences in ingestion rate, body length, and numbers of eggs in the brood pouch of *D. magna* on each sampling day (i.e., day 1, day 3, day 5, and day 7). Measured physiological parameters of *D. magna* on the 7th day were presented as the mean ± *SD* derived from the biological replicates. Because age of *D. magna* can have great effects on the physiological performances as a covariate, two‐way ANOVA (analysis of variance) was used to testing the effects of nutrient limitation (i.e., N‐ and P‐limitation, respectively) on *D. magna* across the entire experiment period. Principal component analysis (PCA) was used to cluster transcriptome samples according to the abundance of genes (Hellinger‐transformed), and difference between two clusters of samples was tested by analysis of similarities (ANOSIM) using the “anosim” function.

## 3 RESULTS

### 3.1 C:N:P of algae as food for *D. magna*

Manipulation of media nutrient contents yielded significantly different elemental composition in *C*. *reinhardtii*. Cells grown in N‐limited medium had the lowest N content (1.95% ± 0.15%, atomic ratio) and higher C:N ratio (16.7 ± 0.1), while cells grown in the P‐limited medium had the lowest P content (0.12% ± 0.01%, atomic ratio) and higher C:P ratio (467.7 ± 89.8), as compared with normal culture (C:N = 7.5 ± 0.2, C:P = 65 ± 6.1, *p* < .01, by Welch two sample *t* test; Table A1).

### 3.2 Physiological characteristics of *D. magna* under different diet conditions

**FIGURE 1:**
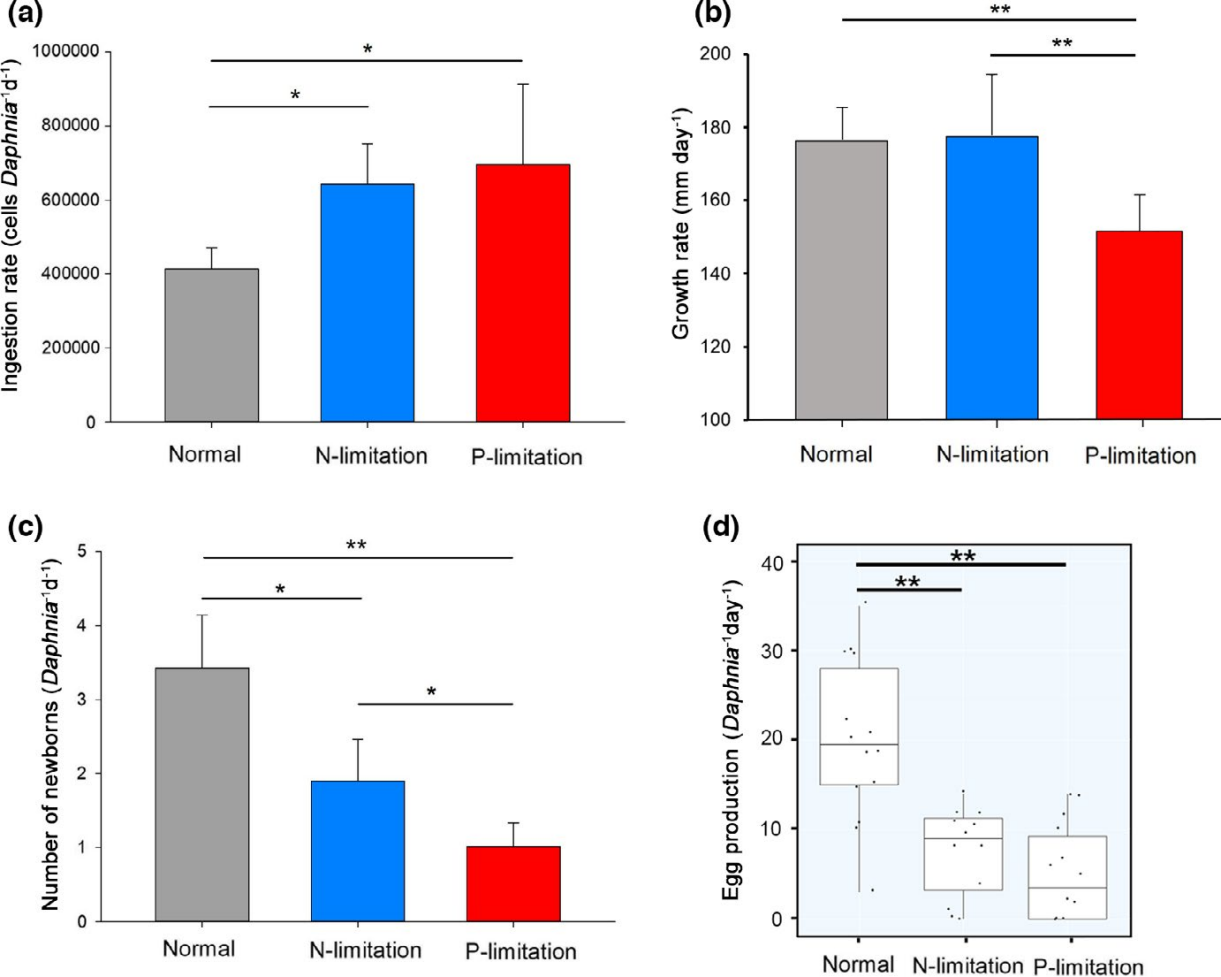
Performances of *Daphnia magna* fed with different types of algae on the 7th day. (a) shows the average (with standard deviation, *SD*) ingestion rate of one individual *D. magna*; (b) shows the average (with *SD*) increase in body length as an index of growth rate for *D. magna*; (c) is the average number (with *SD*) of newborns of one individual *D. magna*; and (d) is the egg production rate of each *D. magna*. Significant difference between groups is marked with asterisk (*p < .05 and **p < .01, by Welch two sample *t* test)

During the 7‐day experiment, *D. magna* with P‐limitation diet had the shortest body length compared to that with N‐limitation or nutrient‐sufficient diet (Figure A1a). After 3 days, *D. magna* exhibited higher ingestion rate but lower egg production rate when fed with N‐ or P‐limitation diet than that with nutrient‐sufficient diet (Figure A1b,c). Significant difference of the physiological performances of *D. magna* between two treatments at each sampling point is shown in Table A2. For the entire experiment period, taking ages into consideration, both N‐ and P‐limitation had significant influences on the ingestion and egg production rates of *D. magna* (Table A3). P‐limitation had a significant effect on the growth rate (i.e., slow increase in body length) of *D. magna*, while this effect was weak for *D. magna* under N‐limitation (Table A3; Figure A1). Here, we focused on the comparison between nutrient‐limited group and nutrient‐sufficient group on the 7th day when transcriptomic samples were collected. As shown in Figure 1, on the 7th day, *D. magna* had higher ingestion rate fed with either N‐limited (64.32 ± 10.83, 10^4^‐cells of algae per *D. magna* per day) or P‐limited algae (69.64 ± 21.7, 10^4^‐cells of algae per *D. magna* per day) than that fed with nutrient‐sufficient (normal) algae (41.22 ± 5.93, 10^4^‐cells of algae per *D. magna* per day; N‐limitation vs. normal: *df* = 3.85, *p* = .0024; P‐limitation vs. normal: *df* = 3.47, *p* = .021, Welch two sample *t* test; Figure 1a). Growth rate of *D. magna* was lower under P‐limitation (151.43 ± 10.17, mm/day) than N‐limitation (177.44 ± 16.93, mm/day) or normal (176.27 ± 9.34, mm/day; Figure 1b). An average of 20 ± 9.00 eggs were produced for each *D. magna* fed with normal algae on the 7th day, which was much higher than *D. magna* fed N‐limited algae (7.58 ± 5.05 eggs per *D. magna*, *df* = 20.98, *p* < .001) and P‐limited algae (5.14 ± 5.43 eggs per *D. magna*, *df* = 21.37, *p* < .001; Figure 1c). Similar to egg production, *D. magna* fed with nutrient‐limited diet had a smaller number of newborns (1.89 ± 0.58 and 1.01 ± 0.32 newborns per *D. magna* with N‐ and P‐limited algae, respectively) compared with nutrient‐sufficient diet (3.43 ± 0.71 newborns per *D. magna*; N‐limitation vs. normal: *df* = 4, *p* = .047; P‐limitation vs. normal: *df* = 4, *p* = .01; Figure 1d).

### 3.3 Transcriptome sequencing and assembly

Transcriptome libraries were constructed using total mRNA and subjected to Illumina deep sequencing (about 18 G per sample). After quality control, clean reads were assembled to contigs: number of contigs in each sample ranged from 227,920 to 306,664, with N_50_ ranged from 1,459 to 1,597 bp. In total, 19,815 unique genes (10,271, on average) of *D. magna* were predicted after sorting and redundancy filtration, representing 89.2% of the total genes in the transcriptome libraries (Table A4).

**FIGURE 2:**
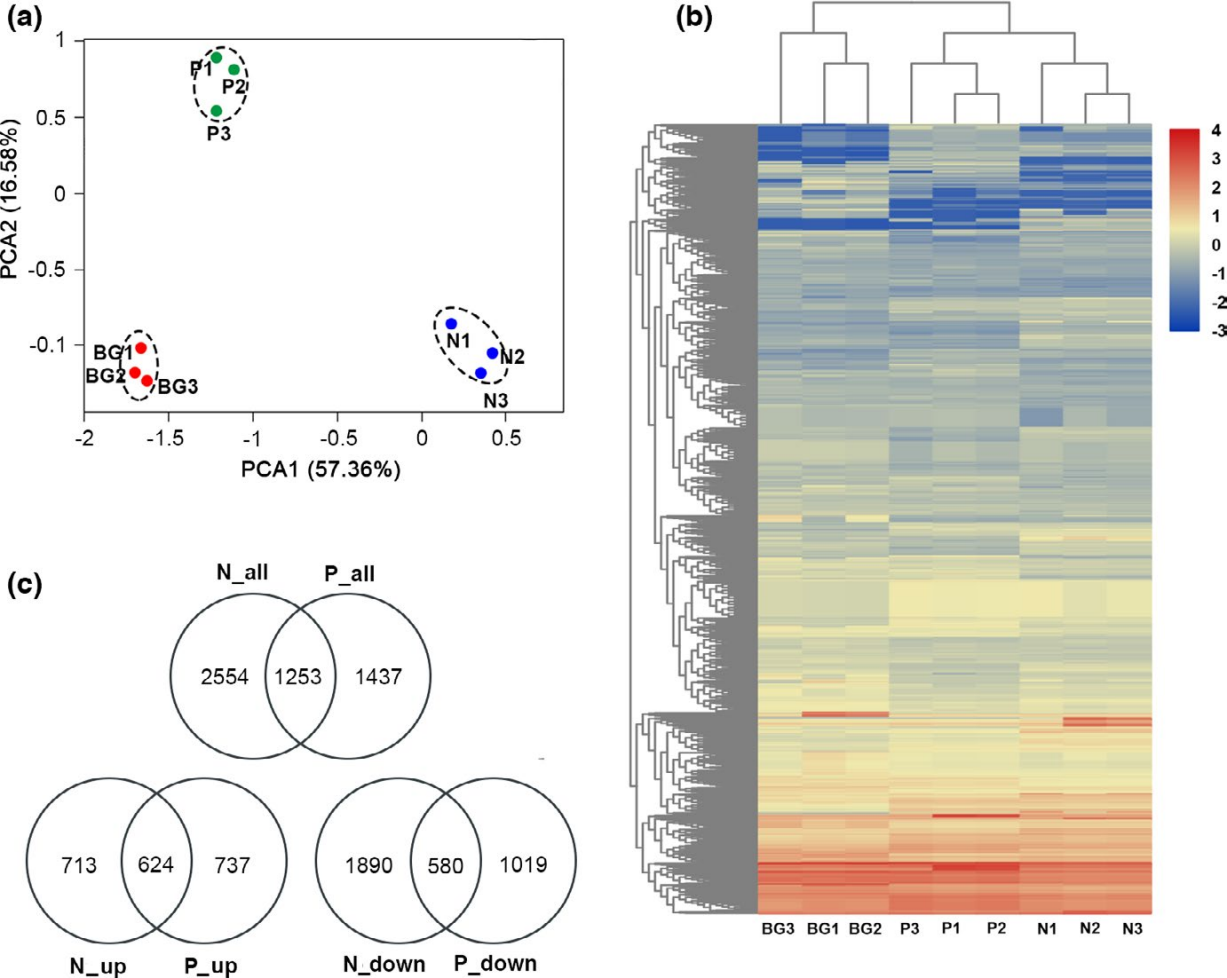
Overview of *D. magna* transcriptome data and gene distributions in each sample. (a) PCA of the RNA‐seq data. Each dot represents one sample. BG is the normal group (i.e., nutrient sufficient). N stands for N‐limitation, and P stands for P‐limitation (numbers 1, 2, and 3 represent triplicates). Samples near to each other are grouped by dashed circles. (b) Heatmap showing the distribution of unique genes in each sample using normalized depths of genes. (c) Venn diagram showing the DEGs in the comparisons among the three treatments

PCA using all unique genes showed that the biological replicates of each treatment (N‐limitation, P‐limitation, and normal) were close to each other and far from other treatments, supporting the reliability of RNA‐seq data. Difference between each experimental group and control group was significant by analysis of similarity (ANOSIM) test (*p* < .01), suggesting the significant effects of low‐quality diet on the gene expression of *D. magna* (Figure 2a,b).

### 3.4 Differential expression analysis

Using a cutoff criteria of |log_2_(fold change)| > 1 and *p* < .05, a total of 3,804 and 2,690 DEGs were identified for *D. magna* under N‐limitation and P‐limitation, respectively, with the normal as comparison. 1,253 DEGs were shared by the two nutrient deficient conditions. With either N‐limitation or P‐limitation diet (compared with normal), more genes of *D. magna* were downregulated (2,470 and 1,599 DEGs, respectively) than upregulated (1,337 and 1,361 DEGs, respectively; Figure 2c).

### 3.5 GO and KEGG enrichment of DEGs

**FIGURE 3:**
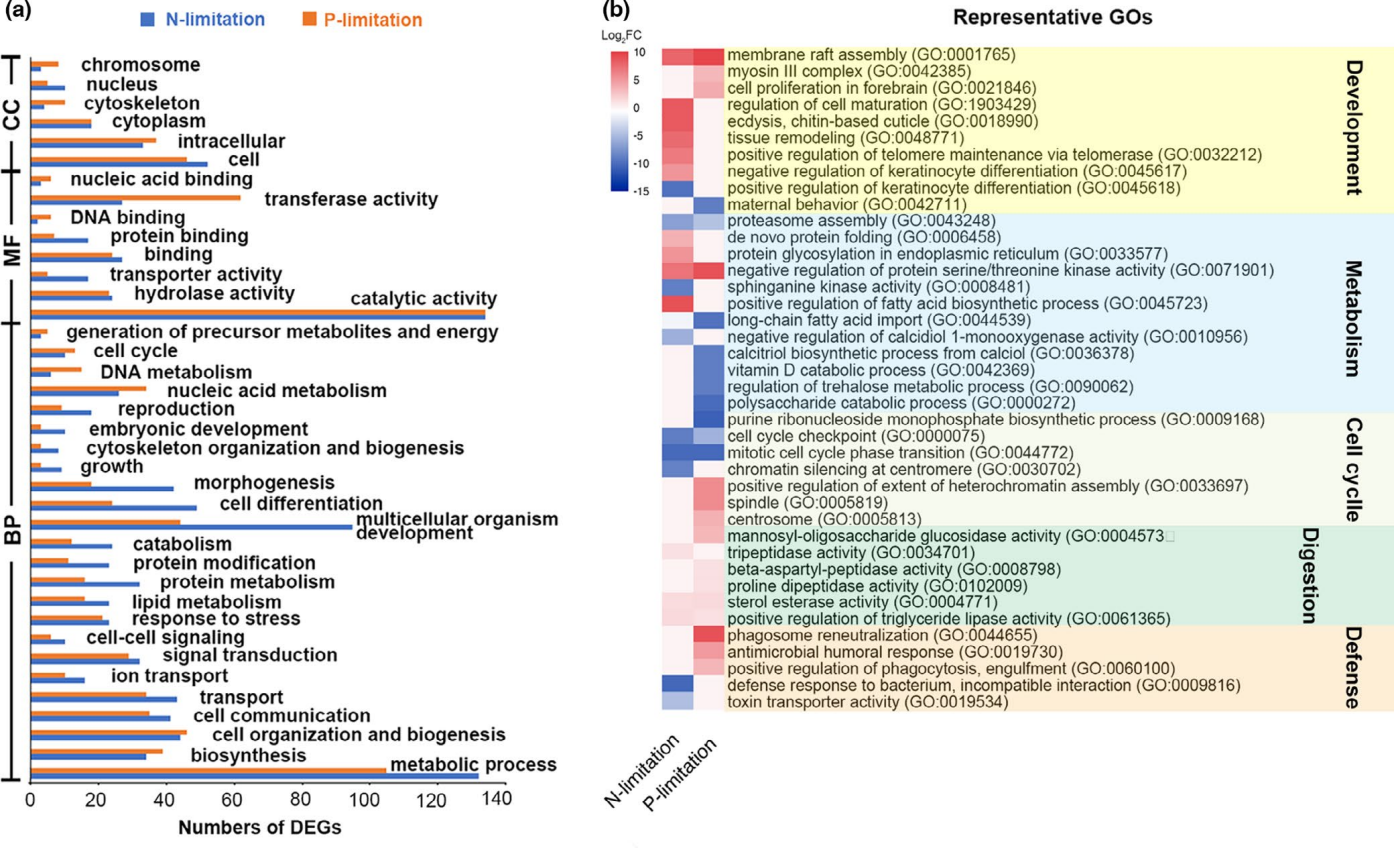
Gene ontology (GO) enrichment of DEGs. (a) GO terms enriched at a high level. BP: biological process, MF: molecular function, and CC: cellular component; (b) heatmap of some representatives of the finest GO terms

All DEGs in this study were assigned to GO terms with three categories: biological process (BP), molecular function (MF), and cellular component (CC). To relate them to the physiology of *D. magna*, we further grouped the GOs at two levels: a higher level (i.e., parent terms; Figure 3a) which is an enrichment category, and the finest level (which is the direct annotation of genes) with some representatives (Figure 3b). For *D. magna* under either N‐limitation or P‐limitation, metabolic process (132 and 105 DEGs, respectively) and catalytic activity (both 134 DEGs) were highly enriched. Notably, much more DEGs were affiliated to multicellular organism development (GO:0007275) in N‐limitation than P‐limitation. For instance, several GOs related to body development, for example, regulation of cell maturation (GO:19034214), ecdysis (GO:0018990), and tissue remodeling (GO:0048771), were highly upregulated (log_2_FC > 4) in the condition of N‐limitation, but not significantly changed under P‐limitation.

**FIGURE 4:**
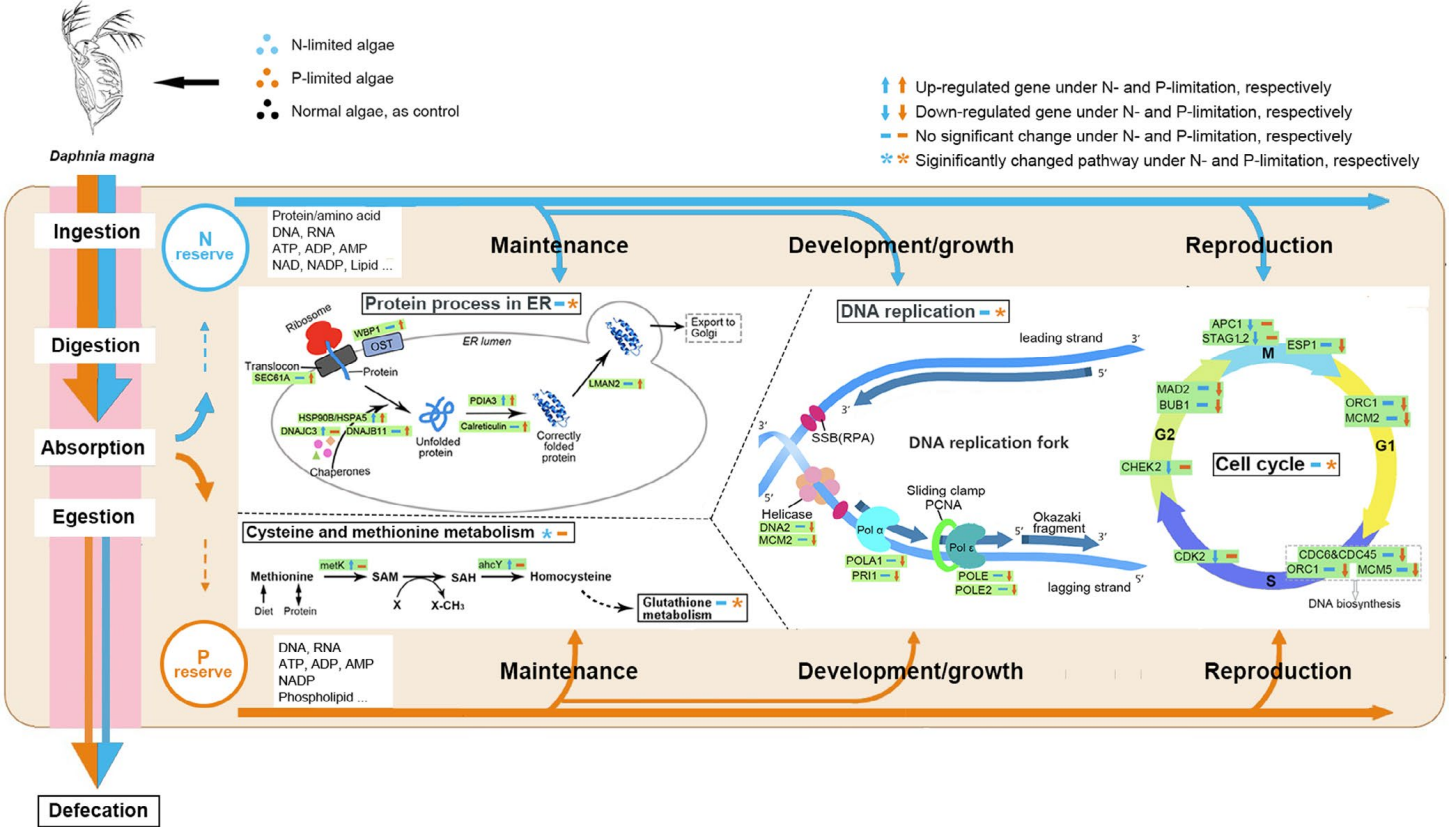
Framework showing the biological pathways of *D. magna* influenced by low‐quality diet. Incoming food from environment is ingested, digested, and assimilated into two nutrient reserves (N and P) which will support the performance (maintenance, growth, and reproduction) of *D. magna*. Five significantly enriched KEGG pathways are illustrated to explain the changes in the performance of *D. magna* under nutrient constrains, with names of pathways in black solid lines. DEGs (some representatives) within these pathways were in green boxes. Detailed information of these DEGs is shown in Table A5. ER: endoplasmic reticulum; OST: oligosaccharyltransferase complex; SAM: S‐adenosylmethionine; SAH: S‐adenosylhomocysteine; SSB: single‐stranded DNA‐binding proteins; RPA: replication protein A; and Pol α (or ε): DNA polymerase α (or ε). G1, S, G2, and M are the four stages of cell cycle

KEGG enrichment of DEGs showed that three pathways (cysteine and methionine metabolism, retinol metabolism, and drug metabolism) were significantly enriched for *D. magna* under N‐limitation (*q*‐value < 0.05; Table 1). In the pathway of cysteine and methionine metabolism, genes of *metK* and *ahcY*, which encode S‐adenosylmethionine synthetase (EC:2.5.1.6) and adenosylhomocysteinase (EC:3.3.1.1), respectively, were upregulated (Figure 4). For *D. magna* under P‐limitation, pathways of DNA replication, cell cycle, glutathione metabolism, and protein processing in endoplasmic reticulum (ER) were significantly enriched (*q* < 0.05). DEGs involved in DNA replication and cell cycle were all downregulated (*n* = 6 and 7, respectively), while DEGs involved in protein processing in ER were all upregulated (*n* = 9).

**TABLE 1:** Enriched pathways of *D. magna* fed by N‐ or P‐limitation diet

KEGG pathway	No. of DEGs (up, down)	*q*‐value
N‐limitation versus normal		
Cysteine and methionine metabolism	10 (3, 7)	0.0019
Retinol metabolism	4 (1, 3)	0.044
Drug metabolism	6 (2, 4)	0.045
P‐limitation versus normal		
Protein processing in ER	9 (9, 0)	0.006
DNA replication	6 (0, 6)	0.049
Cell cycle	7 (0, 7)	0.001
Glutathione metabolism	6 (3, 3)	0.048

Note

Normal: nutrient‐sufficient diet. ER, endoplasmic reticulum. Only pathways with corrected Benjamini *q*‐value < 0.05 were listed here as significantly enriched by differentially expressed genes.

### 3.6 Validation of genes by qPCR

To validate the DEGs identified in the RNA‐seq data in this study, the expression of seven selected DEGs (see detailed information in Tables A5 and A6), which were mainly from enriched KEGG pathways, was confirmed by qPCR. As shown in Figure A2, all qPCR results were in agreement with the RNA‐seq analysis (Spearman correlation *r* = .7, *p* < .05). For example, under N‐limitation, expression of genes encoding S‐adenosylmethionine synthetase (*metK*) and adenosylhomocysteinase (*achY*) increased significantly compared with the normal group (both *p* < .05, Welch two sample *t* test).

## 4 DISCUSSION

### 4.1 Effects of low‐quality diet on *D. magna* physiology: why the effect of P‐limited diet is more severe?

Our results were in accordance with previous studies reporting the effects of low‐quality diet on the growth and reproduction of daphnids, with a conclusion that phosphorus‐limited algae is a much poorer food for *D. magna* (Sterner et al., 1993; Weers & Gulati, 1997). Firstly, we found that growth rate of *D. magna* with N‐limited diet did not change significantly compared with normal. As proteins form the main nitrogen pool in metazoans (~80%), stoichiometric compositional changes associated with N‐limitation should be mostly concentrated in the metabolism of amino acid, and therefore, the profile of amino acid could be an important indicator of the nutritional status and closely related to the performance of zooplanktons (Francois et al., 2016; Wagner et al., 2017). Our result showed that cysteine and methionine metabolism was the most enriched pathway of *D. magna* under nitrogen deficiency. One of the upregulated genes was *metK*, encoding S‐adenosylmethionine synthetase (EC: 2.5.1.6) which produces S‐adenosylmethionine (SAM) from methionine. SAM is the principal biological methyl donor made in the cytosol of every cell, including methylation of proteins, nucleic acids, and lipids. SAM is also required in other important processes, including synthesis of polyamines which are essential for various cellular functions affecting growth and development (Hayashi et al., 2018). Another upregulated gene was adenosylhomocysteinase (*achY*, EC:3.3.1.1) which hydrolyzes S‐adenosylhomocysteine (SAH) to homocysteine (Hcy). The overexpression of the two genes (*metK* and *achY*, from both qPCR and RNA‐seq results) would increase the ratio of SAM:SAH which is frequently used as an indicator of cellular methylation capacity (higher ratio means higher capacity; Caudill et al., 2001). Methylation is an important way for *Daphnia* to response and adapt to environmental stress (Asselman et al., 2017). It is also closely related to cell growth and tissue differentiation of metazoans and could be induced by nutrient restriction (Kusari et al., 2017). Therefore, in our study, the increased degradation of methionine and SAM: SAH ratio may promote the methylation level and stimulate the development (or growth) of *D. magna* with N‐limited diet.

Methionine is necessary for the increase in fecundity of arthropods (e.g., fruit fly and copepods) and plays an important role in controlling the lifespan of animals (Grandison et al., 2009). Methionine is easy to be degraded during the hydrolysis process. Therefore, decrease of methionine, at the same time, may lead to the reduced reproduction of *D. magna* under N‐limitation in our study.

Secondly, *D. magna* fed with P‐limited algae had the lowest growth rate and fewest number of newborns in our study, supporting that P‐limitation causes much severer damage than N‐limitation for daphnids (Sterner et al., 1993; Weers & Gulati, 1997). Our results showed that DEGs (annotated by KEGG) involved in DNA replication and cell cycle were all downregulated in *D. magna* fed with P‐limited diet. This was in accordance with a previous study where genes related to nuclear structure, replication, recombination, and repair were downregulated for daphnids fed with low‐P diet (Roy Chowdhury et al., 2015). Inhibition of P‐limitation on nucleic acid metabolism and cell division could strongly affect the growth rate of phytoplankton, yeast, and bacteria (Brauer et al., 2008; Feng et al., 2015; Vaulot et al., 1996). Thus, our result suggested similar mechanisms may exist in zooplankton, leading to low growth rate of daphnids’ population.

Cell cycle in metazoans is controlled by a number of mechanisms on the gene level. Effects and mechanisms of nutritional limitation on cell cycle and growth rate were well demonstrated for phytoplankton and yeast in previous studies (Brauer et al., 2008; Vaulot et al., 1996), while little is known about zooplankton. For instance, the decrease of growth rate was closely related to the number of cells arrested at the G0/G1 phase for yeast (Brauer et al., 2008) and G2 + M phase for marine diatom *Thalassiosira pseudonana* (Claquin et al., 2002). In our study, for *D. magna* under P‐limitation, downregulated genes within cell cycle were detected linking to all 4 phases of cell cycle, which may explain why daphnids under P‐limitation usually grow much slower. Although several genes (*CHEK2*, *APC1,* and *STAG1*) of *D. magna* under N‐limitation were downregulated in the G2 and M phase, growth rate did not reduce, suggesting the different effects and mechanisms of nitrogen limitation between zooplankton and phytoplankton (Francois et al., 2016). Though several genes within the cell cycle pathway were downregulated under N‐limitation as well, none DEGs co‐occurred in this pathway under both conditions (N‐ and P‐limitation). The same pattern was also observed in the pathway of DNA replication, suggesting the distinct effects and regulation mechanisms between N‐limitation and P‐limitation for *D. magna*.

### 4.2 How do *Daphnia* deal with stoichiometric constrains? Lessons from transcriptomic data

We employed a modified framework of the dynamic energy budget model (Sperfeld et al., 2017) to track metabolic pathways of elemental nutrients from the aspects of maintenance, growth, and reproduction. To investigate how *D. magna* deal with diet‐induced elemental constrains, we focused on the investment of nutrients to the three above aspects and related molecular metabolisms. We mainly focused on the genes within the significantly enriched pathways although other central pathways (e.g., carbon metabolism) are important and essential in dealing with nutrient constrains for daphnids.

Increasing in feeding rate, which is also called compensatory feeding, is one strategy for herbivorous zooplankton to deal with specific nutritional constrains in diet. While previous study showed that daphnids increased their ingestion rate when fed low‐quality algae (Plath & Boersma, 2001), no significant difference in feeding rates between daphnids fed P‐sufficient and P‐depleted algae was also reported (DeMott et al., 1998). These contrasting results suggest that compensatory feeding varies with changes in diet including food abundance, digestibility, and the elemental ratio in food. In our study, ingestion rate of *D. magna* fed with either N‐limited or P‐limited algae was higher on the 7th day than that fed with normal diet, indicating the compensatory feeding of *D. magna* under nutrient constrains. The results were the same after taken body size into consideration (dividing ingestion rate by body length). The increased ingestion rate of *D. magna* under P‐limitation (or N‐limitation) could be reflected in the upregulation of genes related with digestion, such as tripeptidase activity (GO:0034701) and sterol esterase activity (GO:0004771), which is an important mechanism of physiological adaptation of daphnids to nutritionally changing environments (Koussoroplis et al., 2017). Besides, expression of alkaline phosphatase (APA, GO:0004035, a biomarker for P‐limitation in zooplankton) was upregulated under P‐limitation (Log_2_FC = 1.004, *p* < .001, not shown in figures due to its relatively lower fold change value), suggesting the accelerated P acquisition by *D. magna*.

Transcriptomic data allow us to investigate the pathways involved in sequestering limiting elements and the trade‐off strategy of zooplankton under nutrient stress (Jeyasingh et al., 2011). As discussed above, upregulation of genes involved in methionine metabolism explained why growth rate of *D. magna* under N‐limitation kept similar to normal ones. This suggests that N (e.g., amino acid and protein) absorbed from diet could be mainly allocated to the development of somatic tissues of *D. magna* under N‐limitation, rather than reproduction or maintaining the fitness of body (e.g., defense to bacteria and toxin transportation). Meantime, the increased ecdysis (GO:0048771) is not only a necessary step for arthropods to have a lager body size but also contributes to the balance of nutrient after taking in excessive low‐quality food (a way to discharge excess carbon; Hessen & Rukke, 2000).

We showed that under N‐limitation, the highly upregulated genes (log_2_FC > 3, in GO terms) in metabolisms of *D. magna* were related to amino acids and protein (e.g., proteasome assembly, de novo protein folding, and ornithine metabolic process) which are necessary for the body development of zooplankton. We attributed the unaffected growth rate of *D. magna* under N‐limitation to the high methylation level supported by methionine degradation. Thus, for *D. magna* under N‐limitation, the assimilated nitrogen should be mainly assigned to body growth (i.e., development), rather than reproduction. This result is in agreement with the report that copepods under N‐limitation were unable to utilize dietary N efficiently for egg production due to the N demands for maintenance (including growth; Kuijper et al., 2004).

Under P‐limitation, although both growth rate and reproduction rate decreased significantly, a remarkable number of upregulated genes of *D. magna* were detected, which may represent the mechanisms in dealing with phosphorus constrains. Regulation of protein process in ER is one of the post‐translational modifications (PTMs, e.g., phosphorylation, monoubiquitination, and glycosylation) which are important ways for organisms to enhance nutrient acquisition and utilize efficiency (Plaxton & Shane, 2018). In our study, within the pathway of protein process in ER, all DEGs were upregulated under P‐limitation. These genes were mostly distributed across the whole process of protein folding (from entering ER to correctly folded and exported to Golgi), coding the Sec61 complex (*Sec61A*), heat shock proteins (*HSP90*), calreticulin (*CALR*), and intracellular lectins (*LAMN2*; Rapoport, 2007). Our results reflected the accelerated process of protein folding and transporting under phosphorus constrain, without any DEGs detected in the pathway of unfolded protein response. This is different from the transcriptomic response of *Daphnia* to toxic algal diet where unfolded proteins were accumulated and unfolded protein response was activated to keep protein homeostasis.

Several highly expressed genes of *D. magna* (log_2_FC > 3, e.g., membrane raft assembly GO:0001765, myosin III complex GO:0042385, and microtubule GO:0005874) can be related to the accelerated process of protein process in ER and indicate the enhanced maintenance of fundamental structure and functions of cells under P‐limitation. For instance, membrane ensures cell survival upon nutritional stress in eukaryotes. P‐starvation can induce membrane remodeling (e.g., replace phospholipid in membrane by non‐P lipid) and recycling in phytoplankton as a way to save and accumulate phosphorus (Shemi et al., 2016). The upregulation of genes within post‐translation process and cell structure (e.g., membrane) biogenesis was also mentioned in a study comparing ancient and modern daphnids under P‐limitation (Roy Chowdhury et al., 2015). Nevertheless, the underlying P‐related molecular pathways and gene regulation mechanisms need further investigation.

A recent study showed that low‐quality diet (cyanobacteria) could reduce the output of *Daphnia* gut parasites (Manzi et al., 2020). Besides, food with high C:P ratios can significantly reduce bacterial infection rates in daphnids (Frost et al., 2008). Similarly, in our study, genes of *D. magna* related with bacterial infection (e.g., antimicrobial humoral response GO:0019730, and positive regulation of phagocytosis GO:0060100) were highly upregulated (log_2_FC > 3) under P‐limitation. This could be related to the significantly enriched pathway of glutathione (GSH) metabolism in *D. magna* under P‐limitation, as GSH plays an important role in the resistance of organisms to different biotic challenges (Hernández et al., 2017). In contrast, the highly downregulated genes of defense response to bacterium (GO: 0009816, log_2_FC = −10.34) and toxin transporter activity (GO:0019534, log_2_FC = −4.74), together with a significant change in pathway of drug metabolism (four of six DEGs were downregulated), suggested decreased immune responses of *D. magna* under N‐limitation. It is reasonable because immune responses require high quantities of N in forms of proteins to defend against infection (Frost et al., 2005). These results suggest that *D. magna* invested most of the ingested N to body growth (or development) instead of keeping fitness under N‐limitation.

Overall, in this study, using transcriptomic data, we investigated the metabolic mechanisms regulating the physiological changes of *D. magna* under nutrient stress caused by low‐quality diet. We attributed the increased ingestion rate (i.e., compensatory feeding) of *D. magna* under nutrient limitation to the overexpression of genes encoding digestive enzymes. We found that severe negative impacts of P‐stress on *D. magna* (e.g., low growth and reproduction rates) can be explained by the downregulation of genes corresponding all phases of cell cycle. By tracking metabolic pathways of elemental nutrients from the aspects of grazing, maintenance (e.g., post‐translational modification), growth, and reproduction, we showed how some key genes regulated these biological processes that are important to the demography of both zooplankton and phytoplankton and ecosystem functions. However, the genes (and involved pathways) focused in our analysis are a very small percentage of the total number of the annotated genes. Other enriched metabolic pathways in our study, such as retinol metabolism which is essential for the reproduction of zebrafish and mammals (André et al., 2014), are well studied and proven important for the development and homeostasis maintenance of vertebrates but remain unclear for most invertebrates. Their relationships with the phenotypic responses of *D. magna* to nutrient stress require further investigation.

## CONFLICT OF INTEREST

All authors declare no conflict of interest.

## AUTHOR CONTRIBUTIONS

**Zhimeng Xu:** Formal analysis (lead); Validation (equal); Visualization (lead); Writing‐original draft (lead). **Yingdong Li:** Conceptualization (equal); Methodology (equal); Resources (equal); Software (equal); Validation (equal); Writing‐review & editing (equal). **Meng Li:** Supervision (equal); Writing‐review & editing (equal). **Hongbin Liu:** Conceptualization (equal); Funding acquisition (lead); Project administration (lead); Supervision (lead); Writing‐review & editing (equal).

## Supporting information

Appendix S1

## Data Availability

Transcriptome sequencing data were deposited in GenBank (Sequence Read Archive) and are available under the BioProject PRJNA597965.
