# Supplementary material for: Transcriptomic response of Daphnia magna to nitrogen‐ or phosphorus‐limited diet
Source: Ecol Evol. 2021 Jul 16;11(16):11009–19. doi: 10.1002/ece3.7889 (PMC8366849; doi:10.1002/ece3.7889)
Supplement: Supplementary file 1 — Appendix S1 [file ECE3-11-11009-s001.docx]

**Appendix A:**

**
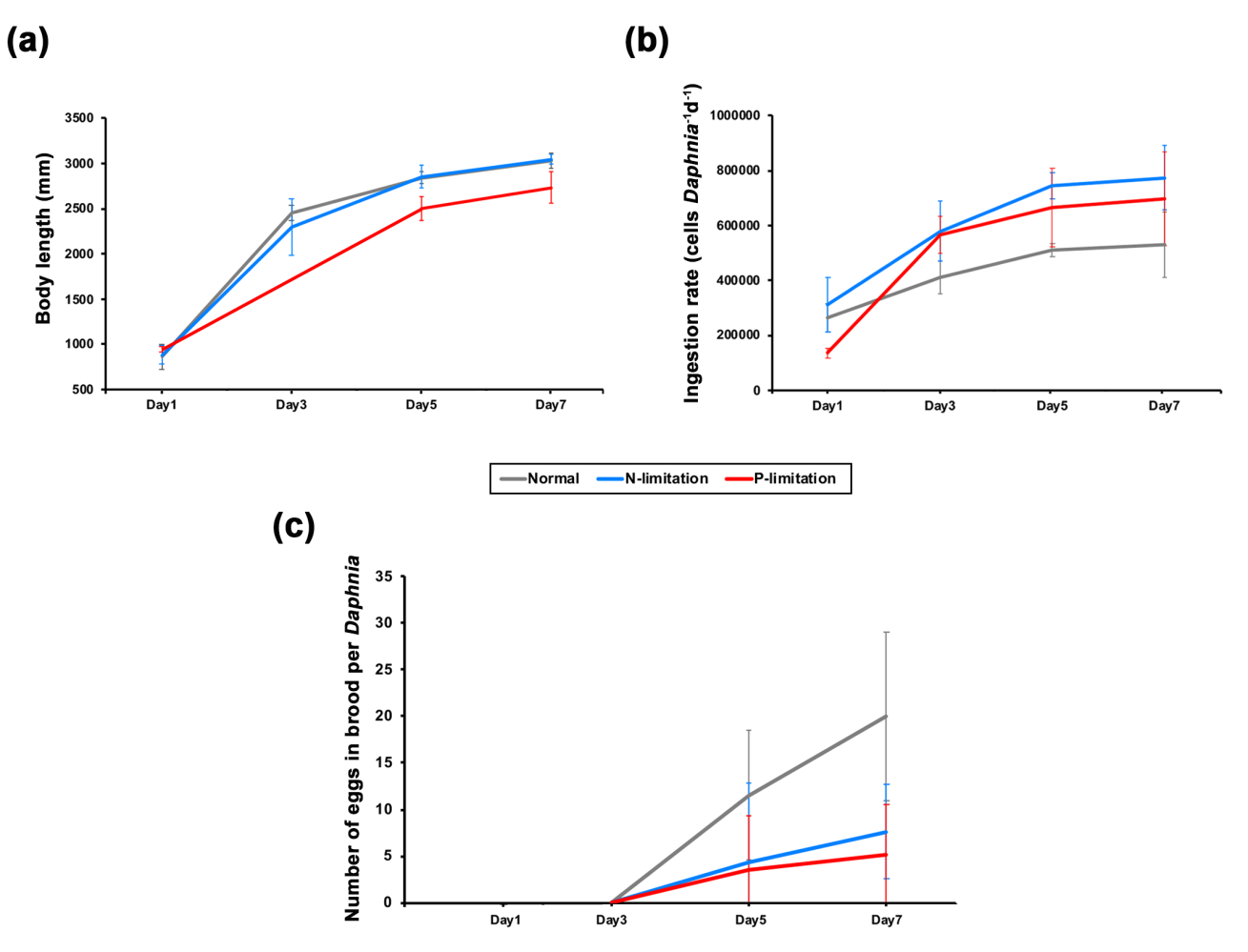
**

**Figure A1** | **Performances of *Daphnia magna* fed with three types of algae (normal, N-limitation and P-limitation) during the experiment.** For *D. magna* under each diet condition, **(a)** shows the average body length of *D. magna* with standard deviation (SD); **(b)** shows the average (with SD) ingestion rate of one individual *D. magna* per day ;**(c)** shows the average number (with SD) of eggs in the brood of one individual *D. magna*. Same colors (grey: Normal diet, i.e., nutrient sufficient diet; blue: N-limitation diet; red: P-limitation diet) are shared by (a), (b) and (c). Significant difference between groups on each sampling day is shown in Table A2.

**
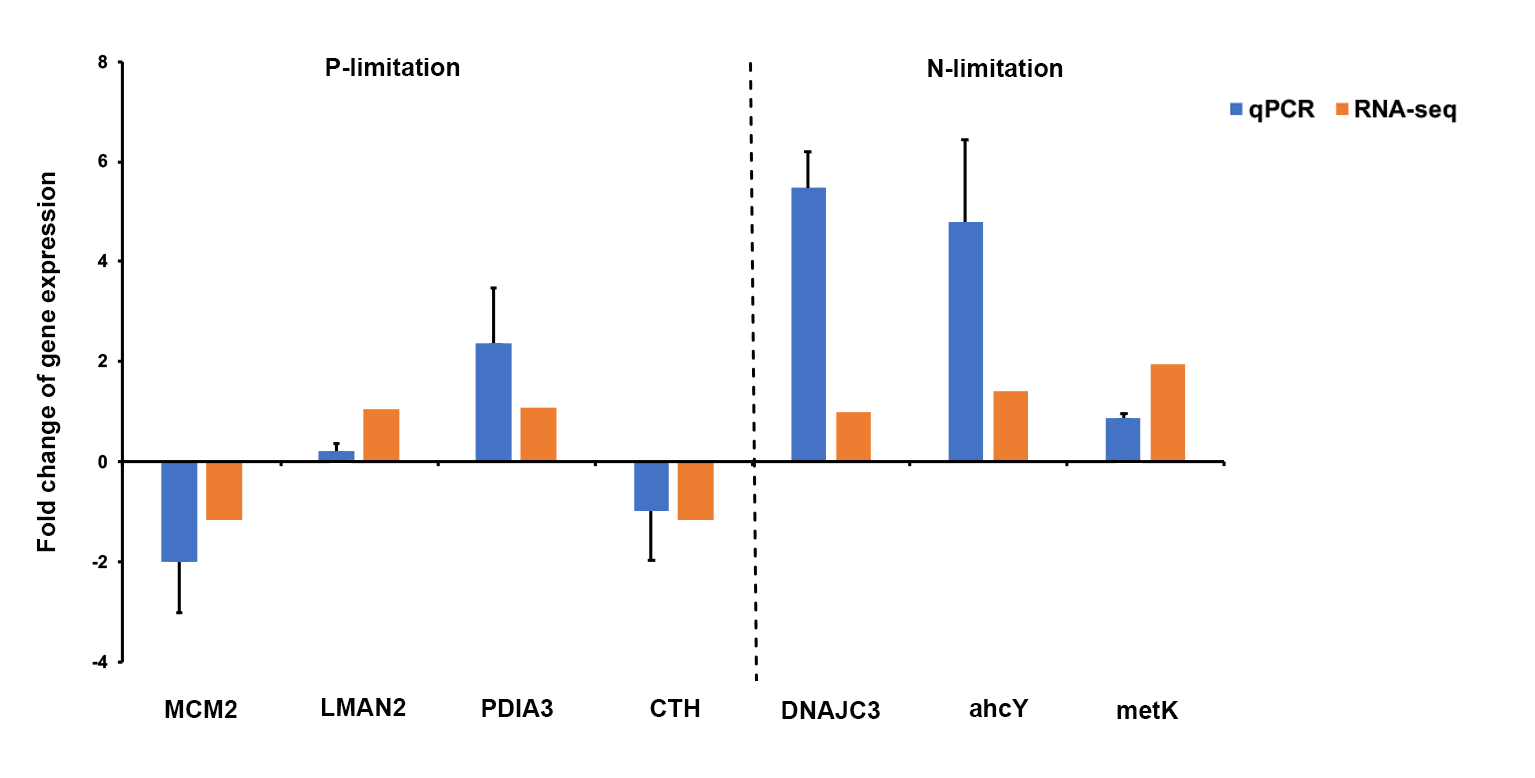
**

**Figure A2 | Validation of RNA-seq data using qPCR.** Comparison of the relative expressions of selected 7 genes of D. magna with N- or P-limitation diet (compared to nutrient sufficient diet) were made between mRNA sequencing (RNA-seq) and qPCR results, with log_2_(Fold change) transformation. All qPCR results were in agreement with the RNA-seq analysis (Spearman correlation r = 0.7, *p* < 0.05).

**Table A1. Elemental ratios (atomic) of *Chlamydomonas reinhardtii* grown under Normal, N-limitation and P-limitation conditions.**

| ***C. reinhardtii*** | **%C** | **%N** | **%P** | **C : N** | **C : P** | **N : P** |
| --- | --- | --- | --- | --- | --- | --- |
| **Normal** | 39.98 ± 1.20 | 5.33 ± 0.48 | 1.83± 0.40 | 7.5 ± 0.20 | 65.0 ± 6.10 | 8.67 ± 0.20 |
| **N-limitation** | 32.57 ± 1.50 | 1.95 ± 0.15 | 1.26 ± 0.10 | 16.7 ± 0.10 | 74.3 ± 8.70 | 4.45 ± 0.10 |
| **P-limitation** | 59.75 ± 2.90 | 3.88 ± 0.89 | 0.12 ± 0.01 | 15.4 ± 0.30 | 467.7 ± 89.80 | 30.3 ± 3.00 |

Normal: nutrient sufficient BG11 medium.

**Table A2. Effects of nutrient limited diet on the daily physiological performances of *Daphnia magna*.**

|  |  | **Day1** | | **Day3** | | **Day5** | | **Day7** | |
| --- | --- | --- | --- | --- | --- | --- | --- | --- | --- |
|  |  | **df** | ***p*** | **df** | ***p*** | **df** | ***p*** | **df** | ***p*** |
| **N-limitation** | |  |  |  |  |  |  |  |  |
|  | **Ingestion rate** | 3.99 | **0.003** | 3.1 | **0.046** | 3.15 | **0.023** | 3.85 | **0.0024** |
|  | **Body length** | 9.17 | 0.79 | 7.62 | 0.29 | 19.25 | 0.86 | 9.86 | 0.7 |
|  | **Eggs** | — | — | — | — | 21.14 | **0.027** | 20.98 | **<0.001** |
|  | **Newborns** | — | — | — | — | — | — | 4 | **0.047** |
| **P-limitation** | |  |  |  |  |  |  |  |  |
|  | **Ingestion rate** | 2.56 | **0.035** | 3.96 | **0.04** | 2.68 | 0.074 | 3.47 | **0.021** |
|  | **Body length** | 6.9 | 0.2 | — | — | 23.48 | **<0.001** | 9.36 | **0.0026** |
|  | **Eggs** | — | — | — | — | 26.73 | **0.002** | 21.37 | **<0.001** |
|  | **Newborns** | — | — | — | — | — | — | 4 | **0.01** |

Student’s *t*-test (Welch two sample *t*-test) was used to test the significant difference between two groups (xxi.e., N- or P-limitation diet vs. nutrient sufficient diet) with “df” representing degree of freedom. Significant data (*p* < 0.05) is indicated in bold. Symbol “—” means lack of data. No eggs or newborns were observed before or on the 3^rd^ day.

**Table A3. Effects of nutrient limitation and experiment time on the physiological performances of *Daphnia magna*.**

| **Response** | **Effect** | **N-limitation** | | |  | **P-limitation** | | |
| --- | --- | --- | --- | --- | --- | --- | --- | --- |
|  |  | **df** | **F** | ***p*** |  | **df** | **F** | ***p*** |
|  | Nutrient | 1 | 39.75 | **<0.001** |  | 1 | 11.73 | **0.002** |
| **Ingestion rate** | Time | 5 | 9.46 | **<0.001** |  | 5 | 161.76 | **<0.001** |
|  | Nutrient × Time | 5 | 9.02 | **<0.001** |  | 5 | 13.76 | **<0.001** |
|  |  |  |  |  |  |  |  |  |
|  | Nutrient | 1 | 62.59 | 0.15 |  | 1 | 175.51 | **<0.001** |
| **Body length** | Time | 3 | 692.99 | **<0.001** |  | 3 | 659.66 | **<0.001** |
|  | Nutrient × Time | 3 | 2.52 | 0.06 |  | 2 | 13.97 | **<0.001** |
|  |  |  |  |  |  |  |  |  |
|  | Nutrient | 1 | 23.68 | **<0.001** |  | 1 | 38.04 | **<0.001** |
| **Eggs** | Time | 2 | 24.24 | **<0.001** |  | 1 | 23.48 | **<0.001** |
|  | Nutrient × Time | 2 | 4.76 | **0.012** |  | 2 | 8.26 | **<0.001** |
|  |  |  |  |  |  |  |  |  |

Two-way ANOVA test was used to test the nutrient effects from nutrient limitation (i.e., N-limitation and P-limitation) on the performances (i.e., ingestion rate, body length and egg production) of *D. magna*, with time (representing the age of *D. magna*) as a covariate. df = degrees of freedom. Significant effect (*p* < 0.05) is in bold.

**Table A4. Assembly information of transcriptome data.**

|  | **Reads No.** | |  | **Contigs information** | | | | | |
| --- | --- | --- | --- | --- | --- | --- | --- | --- | --- |
|  | **Raw** | **Clean** |  | **Total No.** | **Total** | **Av. Length** | **N_50_** | **Longest contig** | ***D. m.* genes** |
|  |  |  |  |  | **base** |  |  |  |  |
| **BG1** | 178776978 | 178727870 |  | 256783 | 1.70×10^8^ | 660 | 1459 | 38443 | 10280 |
| **BG2** | 156749216 | 156706280 |  | 267350 | 1.80×10^8^ | 680 | 1516 | 47606 | 10265 |
| **BG3** | 207102350 | 207045534 |  | 235527 | 1.80×10^8^ | 748 | 1557 | 46108 | 10851 |
| **N1** | 188350584 | 188297762 |  | 284626 | 2.00×10^8^ | 694 | 1597 | 50143 | 9911 |
| **N2** | 176307328 | 176258290 |  | 267377 | 1.80×10^8^ | 689 | 1555 | 46585 | 10070 |
| **N3** | 185635678 | 185583964 |  | 261048 | 1.80×10^8^ | 679 | 1549 | 44384 | 10234 |
| **P1** | 200718860 | 200663685 |  | 263938 | 1.70×10^8^ | 659 | 1461 | 49271 | 10359 |
| **P2** | 161130766 | 161086154 |  | 306664 | 2.00×10^8^ | 655 | 1513 | 38887 | 10290 |
| **P3** | 131320774 | 131298448 |  | 227920 | 1.60×10^8^ | 689 | 1462 | 39323 | 10180 |

BG is the normal diet, N and P are the N-limitation and P-limitation diet, respectively (with 3 replicates from 1 to 3). Reads information contains the number (No.) of raw reads and clean reads (after quality control). Contigs information contains the total number of assembled contigs (Total No.), length of total bases (Total base, bp), average length of the contigs (Av. Length, bp), length of the shortest contig at 50% of the total contigs (N50, bp), length of the longest contig (Longest contig, bp), and number of *Daphnia magna* specific genes (*D. m.* genes) using Non-redundant (NR) database which was used for downstream quantification of gene expression.

**Table A5. Detail information of differentially expressed genes of *Daphnia magna* under nutrient limitation.**

| **KEGG pathway** | **DEG** | **KO** | **Encoding protein** | **Reg.-N** | **Reg.-P** |
| --- | --- | --- | --- | --- | --- |
| **Cysteine and methionine metabolism** | | | |  |  |
|  | metK | K00789 | S-adenosylmethionine synthetase | Up | NS |
|  | achY | K01251 | adenosylhomocysteinase | Up | NS |
|  | CTH | K01758 | cystathionine gamma-lyase | Down | Down |
|  | CBS | K01697 | cystathionine beta-synthase | Down | NS |
| **Cell cycle** |  |  |  |  |  |
|  | BUB1 | K02178 | checkpoint serine/threonine-protein kinase | NS | Down |
|  | CDC6 | K02213 | cell division control protein 6 | NS | Down |
|  | ESP1 | K02365 | separase | NS | Down |
|  | MAD2 | K02537 | mitotic spindle assembly checkpoint protein | NS | Down |
|  | MCM2 | K02540 | DNA replication licensing factor | NS | Down |
|  | ORC1 | K02603 | origin recognition complex subunit 1 | NS | Down |
|  | CDC45 | K06628 | cell division control protein 45 | NS | Down |
|  | CDK2 | K02206 | cyclin-dependent kinase 2 | Down | NS |
|  | APC6 | K03353 | anaphase-promoting complex subunit 6 | Down | NS |
|  | CHEK2 | K06641 | serine/threonine-protein kinase | Down | NS |
|  | STAG1_2 | K06671 | cohesin complex subunit SA-1/2 | Down | NS |
| **DNA replication** |  |  |  |  |  |
|  | POLA1 | K02320 | DNA polymerase alpha subunit A | NS | Down |
|  | POLE | K02324 | DNA polymerase epsilon subunit 1 | NS | Down |
|  | POLE2 | K02325 | DNA polymerase epsilon subunit 2 | NS | Down |
|  | MCM2 | K02540 | DNA replication licensing factor MCM2 | NS | Down |
|  | PRI1 | K02684 | DNA primase small subunit | NS | Down |
|  | DNA2 | K10742 | DNA replication ATP-dependent helicase Dna2 | NS | Down |
| **Protein process in endoplasmic reticulum** | | | |  |  |
|  | HSP90B | K09487 | heat shock protein 90kDa beta | Up | Up |
|  | HSPA5 | K09490 | heat shock 70kDa protein 5 | Up | Up |
|  | DNAJC3 | K09523 | DnaJ homolog subfamily C member 3 | Up | NS |
|  | DNAJB11 | K09517 | DnaJ homolog subfamily B member 11 | NS | Up |
|  | CALR | K08057 | calreticulin | NS | Up |
|  | PDIA3 | K08056 | protein disulfide-isomerase A3 | Up | Up |
|  | LMAN2 | K10082 | lectin, mannose-binding 2 | NS | Up |
|  | SEC61A | K10956 | protein transport protein SEC61 subunit alpha | NS | Up |
|  | WBP1 | K12670 | oligosaccharyltransferase complex subunit beta | NS | Up |
|  | SSR2 | K13250 | translocon-associated protein subunit beta | NS | Up |

DEG: differentially expressed gene. Listed DEGs are genes enriched the 5 pathways (*q* < 0.05, see Table 1) discussed in this study. KO: KEGG orthology. Reg.-N: regulation of DEGs in the N-limitation condition. Reg.-P: regulation of DEGs in the P-limitation condition.

**Table A6. The primers of selected gene in qPCR analysis for transcriptomics.**

| **Selected genes** | **Forward primer** | **Reverse primer** | **Length** |
| --- | --- | --- | --- |
| S-adenosylmethionine synthetase (metK) | ATCGCTCTGCTGCTTATG | TTGGATGTGCCGTAATCG | 145 |
| adenosylhomocysteinase (ahcY) | GCGTCAGGAATATCAACCA | CAGGCGAAGATGGAGAAC | 283 |
| cystathionine gamma-lyase (CTH) | CTACCGTCTTCAATCTGCTA | GCCGAGTTCATCACAATATC | 349 |
| DnaJ homolog subfamily C member 3 (DNAJC3) | GCATCCAGACAACTTCCA | TCCTCGCCATTATCATACTT | 129 |
| protein disulfide-isomerase A3 (PDIA3) | CAGTCCTTCACCGATTCTT | CAACAGCAGTCTTCAATGG | 268 |
| lectin, mannose-binding 2 (LMAN2) | ATGCTCGTGATAGAATGGTT | ATAGTATCCTGTCGGTAATCG | 350 |
| DNA replication licensing factor MCM2 (MCM2) | GCAAGGCTATTCAGTAAGTG | GTCTCAACGAACGGATGT | 365 |

Length is the amplicon length of target gene (bp).
